# When Age Is More Than a Number: Acceleration of Brain Aging in Neurodegenerative Diseases

**DOI:** 10.2967/jnumed.125.270325

**Published:** 2025-10

**Authors:** Elena Doering, Merle C. Hoenig, James H. Cole, Alexander Drzezga

**Affiliations:** 1 Forschungszentrum Jülich, Institute of Neuroscience and Medicine–Molecular Organization of the Brain (INM-2), Jülich, Germany;; 2 Department of Nuclear Medicine, Faculty of Medicine and University Hospital, University of Cologne, Cologne, Germany;; 3 Hawkes Institute, Department of Computer Science, University College London, London, United Kingdom;; 4 Dementia Research Centre, Queen Square Institute of Neurology, University College London, London, United Kingdom; and; 5 German Center for Neurodegenerative Diseases, Bonn-Cologne, Germany

**Keywords:** brain age, machine learning, neuroimaging, neurodegeneration, dementia

## Abstract

Aging of the brain is characterized by deleterious processes at various levels including cellular/molecular and structural/functional changes. Many of these processes can be assessed in vivo by means of modern neuroimaging procedures, allowing the quantification of brain age in different modalities. Brain age can be measured by suitable machine learning strategies. The deviation (in both directions) between a person’s measured brain age and chronologic age is referred to as the brain age gap (BAG). Although brain age, as defined by these methods, generally is related to the chronologic age of a person, this relationship is not always parallel and can also vary significantly between individuals. Importantly, whereas neurodegenerative disorders are not equivalent to accelerated brain aging, they may induce brain changes that resemble those of older adults, which can be captured by brain age models. Inversely, healthy brain aging may involve a resistance or delay of the onset of neurodegenerative pathologies in the brain. This continuing education article elaborates how the BAG can be computed and explores how BAGs, derived from diverse neuroimaging modalities, offer unique insights into the phenotypes of age-related neurodegenerative diseases. Structural BAGs from T1-weighted MRI have shown promise as phenotypic biomarkers for monitoring neurodegenerative disease progression especially in Alzheimer disease. Additionally, metabolic and molecular BAGs from molecular imaging, functional BAGs from functional MRI, and microstructural BAGs from diffusion MRI, although researched considerably less, each may provide distinct perspectives on particular brain aging processes and their deviations from healthy aging. We suggest that BAG estimation, when based on the appropriate modality, could potentially be useful for disease monitoring and offer interesting insights concerning the impact of therapeutic interventions.

The common saying that “age is just a number” is often used to comfort those who feel overwhelmed by the seemingly relentless and uncontrollable progress of aging. However, from a biologic standpoint, age is more than a single number, and the biologic aging process may not, indeed, be entirely uncontrollable. As the brain ages, it undergoes profound alterations at various levels, including molecular composition, structural integrity, and functional connectivity. The biologic processes associated with brain development and aging are sequential, nonlinear, and complex. During the first years of life, brain development is associated with cortical maturation and the formation of neuronal connections (1,2). Brain development is strongly accelerated during infancy and early childhood and typically peaks before the age of 30 (1). As opposed to brain development, brain aging is generally seen as the accumulation of deleterious biologic changes during adulthood, resulting in progressive impairment of brain function (3). Regions throughout the brain display differential vulnerability toward the aging process, with brain regions that are believed to develop later also showing signs of earlier degeneration (4). A multitude of factors, including genetic, developmental, environmental, and disease-specific attributes can influence how fast the brain ages, leading to a heterogeneous expression of brain aging. Most people experience some degree of normal age-related changes, including loss or alterations of brain structure and function. Some individuals are able to maintain exceptional cognitive function until highly advanced age, potentially indicative of a slowed brain aging process (so-called super agers (5,6)). On the other hand, patients with specific neurodegenerative diseases, for example, Alzheimer disease (AD), demonstrate profound neurodegeneration and decline in cognitive abilities, which in turn has been associated with accelerated brain aging (7–11). Importantly, brain aging and neurodegeneration are not necessarily synonymous. Although some age-related changes may contribute to neurodegenerative processes, others might occur independently or even provide resilience against neurodegeneration. To further study this, recent advances in data science have allowed the estimation of an individual’s brain age from neuroimaging data using machine learning. Given the tremendous interindividual differences in brain aging, understanding brain age as a potential biomarker, as well as its clinical implications, may advance risk assessment, diagnosis, staging, and monitoring of various age-related neurodegenerative diseases and their therapies.

Brain age models are trained to estimate chronologic age from neuroimaging data on the basis of large datasets of healthy agers, where they learn to identify specific patterns that associate neuroimaging features—such as regional gray matter volume, connectivity, or metabolic activity—with chronologic age. These models summarize age-related changes derived from neuroimaging features, yielding a personalized estimate of brain age. The difference between measured brain age and chronologic age of an individual is called the brain age gap (BAG; sometimes also called brain-predicted age difference or brain-age gap estimation). The BAG is a number representing the deviation from normal aging in years (i.e., the deviation from chronologic age) at a particular point in time and for a particular examined modality (e.g., structural MRI): a positive BAG (>0 y) indicates that an individual’s brain appears older on this modality than a person’s chronologic age, whereas a negative BAG (<0 y) suggests the opposite (Fig. 1). In neurodegenerative disorders, the BAG can be understood as a marker that quantifies pathophysiologic brain changes resembling accelerated aging. That is, if a neurodegenerative process and aging typically affect similar regions of the brain, the BAG can quantify the extent to which the disorder causes an acceleration of the aging process. Moreover, a positive BAG may then also indicate increased susceptibility to additional age-associated neurologic conditions. Although it may be challenging to reduce brain age itself (i.e., to rejuvenate the brain), slowing down an accelerated aging process seems conceivable—meaning it may be possible to reduce the BAG by means of successful therapies against neurodegeneration or neurodegenerative pathology (12,13). As a consequence, the BAG and its potential reduction through intervention not only could be useful for disease monitoring but also could be a valuable readout for clinical trials assessing treatment efficacy.

**FIGURE 1.**
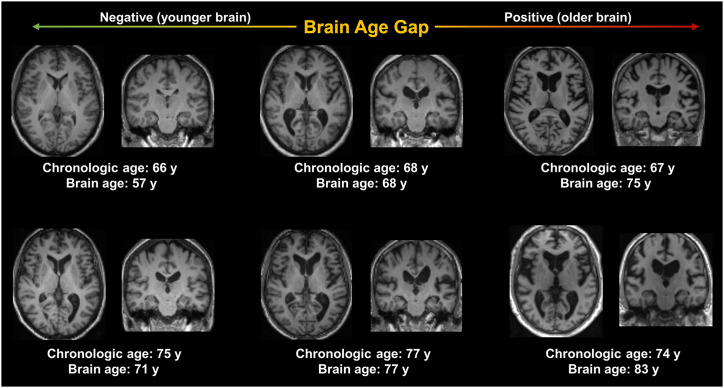
Chronologic and structural MRI brain ages of 6 patients with MCI. Age effects are distinctly apparent on structural MRI, for example, in enlargement of ventricles, as well as general cortical and subcortical atrophy. BAG captures acceleration of brain aging, for example, due to neurodegenerative disease, and can provide useful information for disease and therapy monitoring. Brain age was estimated using our models previously implemented in Doering et al. (19) for our previous publication. Same slice is depicted for all scans.

This article will therefore outline how to compute brain age, how BAGs estimated from different neuroimaging modalities are associated with specific phenotypes of neurodegenerative disease and provide current evidence on the association of BAGs and treatment efficacy.

## COMPUTATION OF BRAIN AGE

**FIGURE 2.**
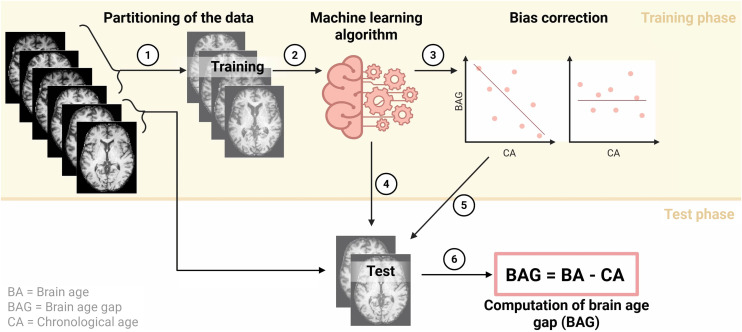
Computation of brain age. Prespecified portion of data (1) is allocated for training of machine-learning regression algorithm (2). Resulting brain age (BA) estimates are known to have inherent bias, which can be eliminated using bias correction (3). Trained machine-learning model can then be applied to test data to yield BA estimate (4). Bias correction parameters inferred during training stage can be applied to model output to obtain bias-free BA estimates (5). Finally, BAG is computed by subtracting chronologic age (CA) from BA (6). Figure created with BioRender.com.

The computation of brain age is accomplished using supervised machine-learning regression models (Fig. 2), whereas the specific pipeline depends on sample size, computational resources, as well as processing time (14). Overall, support vector, relevance vector, and gaussian process regression are popular model types. Usage of convolutional neural networks for this task is on the rise (15–17), due to fewer preprocessing requirements; however, they require larger sample sizes for training and are more computationally expensive.

For each machine-learning task, there are 2 phases, namely the training and the test phase, for which different and independent parts of the data are reserved. Partitioning of the data into training and test sets can be achieved either by allocating a specific percentage of the data to training and testing (e.g., using a 70%–30% split) or by using cross-validation, wherein multiple splits are performed on the data. In the initial training phase, training data of healthy agers, for example, cognitively normal subjects, are provided to the model as input for learning to associate neuroimaging-derived features to an individual’s chronologic age. Healthy agers are used since they allow the model to learn the estimation of a person’s age from their brain scan in the absence of known neurologic abnormalities. For reliable assessment of patients’ brain ages in subsequent analyses, healthy agers used to train and validate the models should cover approximately the patients’ age range. Data can be provided to the model in the form of either voxels, pattern expression scores obtained through principal component analysis, or as specific metrics in a set of brain regions (e.g., average voxel value, cortical thickness, or fractional anisotropy per region) (14). Notably, brain age estimates obtained from such a pipeline yield a bias, wherein the brain age of older individuals is underestimated, and the brain age of younger individuals is overestimated, caused by a regression-to-the-mean effect. Several straightforward bias correction methods have been suggested and were thoroughly discussed by de Lange and Cole (18). Briefly, bias correction can be achieved using residual approaches with or without consideration of chronologic age. Although there is no universally preferred option, it is worth noting that accounting for chronologic age in bias correction increases the similarity between brain and chronologic age, whereas not accounting for chronologic age decreases the similarity, that is, accuracy. Alternatively, explicit bias correction can be avoided, and chronologic age can be used as a covariate in subsequent analyses in which the BAG is the dependent variable. This covariate approach is sometimes preferred as it circumvents the need to choose among competing bias correction methods. Accuracy assessment of the trained model is accomplished by computing the mean absolute error (lower is better, and the optimal value is 0) and other quantitative metrics, for example, the coefficient of determination (*R*^2^; higher is better, and the optimal value is 1), between brain and chronologic age in the test set of healthy agers. A mean absolute error below 5 y is generally considered acceptable when predicting values across the adult life span (14). To compare the performance of different brain age models across studies, *R*^2^ is the preferred choice, as it is unaffected by potentially different age distributions in the test sets. Finally, a reliable brain age estimation pipeline can be applied to compute the BAG of individuals both in another previously untouched test set of healthy agers and in patients with assumed neurologic abnormalities to quantify their magnitude of deviation from healthy aging. Given that aging is a multifactorial phenomenon, BAGs may be estimated from different modalities, with potentially different implications for the divergence from healthy aging.

## DIFFERENT BAGS AND THEIR ASSOCIATION WITH NEURODEGENERATIVE DISEASE

Brain age, or BAGs, have previously been estimated from various modalities, yielding structural, molecular, functional, or microstructural perspectives on brain aging. The most common modality to estimate BAGs in neurodegenerative diseases is structural MRI, which depicts atrophy. Additionally, features from molecular imaging, such as PET, functional imaging, such as resting-state functional MRI (fMRI), and electroencephalography, or microstructural imaging such as diffusion MRI have previously been used to estimate BAGs.

### Structural BAGs from T1-Weighted MRI

BAGs estimated from T1-weighted MRI relate to the deviation from normal structural brain aging, and they have been the most extensively researched thus far. Such structural BAGs can be understood as phenotypic biomarkers; that is, they reflect observable patterns of brain changes associated with aging, rather than directly reflecting underlying cerebral mechanisms. Higher structural BAGs (i.e., older-looking brains) have consistently been associated with more severe cognitive dysfunction (15,19–21) and future progression from mild cognitive impairment (MCI) to dementia (11,15,19). Consistently, structural BAGs have been shown to increase over time in patients clinically diagnosed with AD dementia (20,22). Evidence suggests that elevated structural BAGs may be more specific to AD compared with other neurodegenerative diseases, as they correlate with AD biomarkers and are significantly higher in MCI patients on the verge of progressing to AD dementia compared with those progressing to other types of dementia (such as frontotemporal or Lewy body dementia) (15). The authors speculated that AD may thus mimic an accelerated version of normal structural aging, whereas other types of dementia present with distinct, disease-specific structural damage that makes the brain appear older but does not follow the typical order of age-related structural changes. Unlike existing structural MRI biomarkers for AD, for example, those obtained from individual regions such as the hippocampus, structural BAGs integrate atrophy patterns across the entire brain, potentially capturing AD-related changes in more complex regional (co)variance patterns across the brain. Furthermore, structural BAGs offer an individual marker of deviation from expected aging patterns, allowing for individualized risk stratification beyond established absolute measures of brain structure. In summary, the extant literature provides convincing evidence that structural BAGs may complement prognostic and monitoring assessments for (especially AD) dementia patients. It appears promising to test the added potential of considering structural BAGs alongside, for example, β-amyloid PET for patient stratification in clinical trials (9), as BAGs may capture broad neurodegenerative changes that could aid in risk stratification of early AD cases.

Beyond AD, structural BAGs are also elevated in Parkinson disease (23–25), multiple sclerosis (12,26,27), and some patients with amyotrophic lateral sclerosis (28) compared with healthy agers. In multiple sclerosis patients, structural BAGs increase with disease duration and predict the progression of disability (26,27), suggesting structural BAGs could be useful as a marker of the biologic progression of multiple sclerosis. For Parkinson disease and amyotrophic lateral sclerosis, on the other hand, the association of the BAG and clinical disease parameters was most convincingly shown for cognitive dysfunction, rather than overall symptom severity. Hence, the involvement of structural BAGs in Parkinson disease or amyotrophic lateral sclerosis is possibly restricted to the frequent cooccurrence of dementia in these disorders (29,30), rather than representing an isolated disease mechanism (31).

### Molecular Imaging–Derived BAGs

Molecular imaging techniques, such as PET or SPECT, can be used to visualize the brain’s metabolism, the accumulation of protein pathologies using compound-specific tracers (e.g., for amyloid or tau deposits), or the integrity of neurotransmitter systems (e.g., for dopamine). Unlike structural BAGs, molecular imaging–derived BAGs could capture mechanistic deviations from healthy aging trajectories that may reflect emerging pathophysiologic processes. Several molecular imaging techniques capture age-associated changes and thus might be suitable candidates for brain age estimation. For example, [^18^F]FDG PET studies have shown that brain metabolism declines by ∼12%–13% over the adult life span, most notably in the neocortex (32). Likewise, amyloid accumulation is not exclusive to AD but is also—to some extent—a part of normal aging (33). Even dopamine transporter imaging, typically indicated for the assessment of movement disorders, shows a gradual decrease in striatal binding ratios with healthy aging (34,35). Given that such molecular changes often precede atrophy (36), BAGs estimated from PET or SPECT could theoretically be more sensitive to early-stage neurodegenerative changes than structural BAGs. In practice, however, only a few studies investigated molecular imaging–derived BAGs and their association with neurodegenerative disease, which are summarized here.

Metabolic BAGs were previously derived from [^18^F]FDG PET and found to be positively correlated with structural BAGs; however, they were neither strongly associated with markers of AD pathology nor significantly elevated in individuals progressing from MCI to AD dementia during follow-up (15,19). These findings do not support a primary role for metabolic BAGs in the early detection of AD, possibly because age-related hypometabolism involves primarily the frontal lobes, whereas AD affects the frontal lobe only later on in the disease course (32). Instead, data from Lee et al. (15) indicated that metabolic BAGs might represent a useful biomarker for the timely identification of frontotemporal dementia, as metabolic, but not structural BAGs were significantly increased in MCI–to–frontotemporal dementia progressors, and both BAGs were higher in patients with frontotemporal dementia than in stable controls. Finally, 1 recent study estimated BAGs using [^18^F]flortaucipir, which depicts cerebral tau pathology accumulation (37). The tau-derived brain age was close to chronologic age in cognitively healthy subjects and increased with disease progression and symptom severity of AD, indicating that the model may have identified subtle age-related patterns of tau accumulation that are exacerbated in AD. To the best of our knowledge, to date, no studies exist that estimate brain age from amyloid PET or dopamine PET/SPECT. Taken together, molecular imaging–derived BAGs remain underexplored but hold considerable promise for advancing our understanding of neurodegenerative diseases as potential manifestations of accelerated aging of individual physiologic processes. We encourage future research to systematically assess molecular imaging–derived BAGs across various tracers and clinical populations, given their expected potential for early detection and disease characterization. In addition, evaluating whether structural information can improve molecular imaging–based brain age estimation may be a valuable next step. For example, incorporating partial volume correction as a preprocessing step in brain age models may improve regional signal accuracy, especially in cortical and subcortical gray matter.

### Functional BAGs from fMRI or Electroencephalography

Recent studies have also explored BAGs derived from resting-state fMRI and electroencephalography, which reflect deviations from typical age-related changes in brain function. Functional BAGs, from both fMRI and electroencephalography, have been found to be elevated in AD (38,39) and to a lesser extent in MCI and the behavioral-variant of frontotemporal dementia (38). Higher sleep electroencephalography–derived BAGs were not only found to be associated with greater cognitive dysfunction but also to cooccur with modifiable dementia risk factors—such as a higher apnea–hypopnea index and greater smoking exposure (40). Although studied mostly in cognitively impaired individuals, sleep electroencephalography–derived BAGs may aid early detection, as sleep disturbance tends to be an early indicator of various forms of neurodegeneration (41–43), and the association of sleep electroencephalography–derived BAGs with modifiable risk factors further underscores sensitivity to preneurodegenerative changes. However, further research is warranted for both functional BAGs. For instance, fMRI-based BAGs were not found to be linked to the amount of amyloid or tau pathology in amyloid-positive, cognitively impaired individuals (44), nor in preclinical familial AD (45), suggesting non-AD pathologies may drive fMRI-based functional brain age acceleration, or the latter may be masked by compensatory mechanisms. Moreover, abnormally high fMRI-derived BAGs have been associated with cognitive decline beyond neurodegenerative diseases, such as in patients with epilepsy (46). Thus, the specific mechanisms contributing to accelerated aging of brain function and their potential involvement in neurodegenerative diseases remain to be further elucidated.

### Microstructural BAGs from Diffusion MRI

Finally, some studies estimated BAGs from white matter microstructure data using diffusion-weighted MRI in the context of neurodegenerative diseases. Microstructural BAGs were found to be elevated in initially cognitively normal individuals who later progressed to MCI (47) or increased in clinical dementia ratings (48). Although pending further investigation, these observations are in line with previous studies outlining the early diagnostic value of white matter alterations on diffusion-weighted MRI (49,50), for which microstructural BAGs may be a useful summary marker.

## BAGS AND TREATMENT

### Effect of BAGs on Treatment Outcomes

A recent study by Tseng et al. (51) found that lower baseline structural BAGs were associated with better treatment response to cholinesterase inhibitors in patients with MCI (mean BAG of responders = 3.9 y; mean BAG of nonresponders = 8.4 y) despite comparable cognitive performance at baseline. Similarly, the effectiveness of repetitive transcranial magnetic stimulation in treating depression among patients with AD or vascular dementia was more closely linked to lower baseline structural BAGs than to gray matter volume or to baseline depression or cognitive impairment severity (mean MRI BAG of remitters = −3.0 y; mean MRI BAG of nonremitters = 4.6 y) (52). Notably, the diverging significance of differences in baseline BAGs and clinical scores in the dementia studies demonstrate that the BAG does not always correlate directly with cognitive function, as factors such as cognitive reserve may modulate the impact of age-related brain changes on clinical symptoms. Cognitive reserve refers to the ability of the brain to compensate for ongoing neurodegenerative processes (53,54). As such, individuals with similar BAGs may show markedly different cognitive trajectories, warranting further research on the interplay of cognitive reserve and BAG. Together, current evidence suggests that structural BAGs, as a biologic marker of disease progression, might provide a useful predictive indicator of treatment efficacy in patients with dementia, and they could potentially facilitate patient stratification for clinical trials.

### Effect of Treatment and Intervention on BAGs

To the best of our knowledge, only 1 study has investigated whether treatment can reverse BAGs in neurodegenerative diseases: McMurran et al. (12) reported an 11-mo reduction in structural BAGs after 6 mo of bexarotene therapy in patients with multiple sclerosis. Beyond neurodegenerative diseases, 3 studies further support the notion of BAGs as a potential marker to reflect the effect of treatment or interventions: Chu et al. (55) demonstrated that a single night of sleep deprivation increased an individual’s structural BAG by 1–2 y, which, in turn, was reset to baseline levels after 1 night of recovery sleep. In individuals aged 26–58 y, Wan et al. (13) recently outlined that regular aerobic exercise over the course of 12 mo reduces structural BAGs by approximately 7 mo. Finally, structural BAGs were shown to be reduced by 1.1 y in healthy individuals aged 23–47 y after administration of the antiinflammatory drug ibuprofen (56). Taken together, the current body of literature not only provides compelling evidence for considering (structural) BAGs as an additional indicator of treatment efficiency in clinical trials but also points to the potential sensitivity of BAGs toward lifestyle differences and common medication intake. Such factors should influence the evaluation of the BAG in potential diagnostic or monitoring contexts.

### Availability and Limitations of the BAG

Most neurodegenerative disease trials already include neuroimaging as part of their protocols—commonly used for eligibility screening, stratifying participants by biomarker status, or monitoring disease progression. In such cases, BAG metrics might be considered an attractive candidate for secondary or exploratory endpoints, as they require no additional data acquisition, cost, or participant burden. In these contexts, the BAG does not necessarily need to outperform established biomarkers such as hippocampal volume or regional PET signal to be valuable. By capturing complementary information—such as diffuse or distributed brain changes not adequately represented by conventional regional biomarkers—it may enhance trial readouts or facilitate the identification of responder subgroups (57).

However, BAG metrics also have certain drawbacks, as they may be influenced by modality-specific factors (e.g., image resolution or preprocessing choices) or by the training data and modeling approach (58). Moreover, interpretation of BAGs can be confounded by the model’s accuracy, as well as by factors such as head motion (59), scanner variability (58), and comorbidities (60,61). Furthermore, as outlined above, not every BAG may show strong and consistent associations with clinical outcomes in every disease. This is likely due to the fact that brain age models derived from neuroimaging data of healthy agers predominantly capture deviations in typical age-related patterns. Thus, they may have limited sensitivity to pathologic changes in regions other than those typically affected by aging. Finally, current imaging-based brain age models may not capture upstream mechanisms such as inflammation, proteostasis failure, or epigenetic changes, as they are not currently measurable by imaging. As such, whereas easily implemented, BAGs should be interpreted with awareness of their context-specific strengths and limitations.

## CONCLUSION

In this article, we outlined that brain age is more than a number; there are multiple brain ages derived from different modalities, and they represent measurable and potentially modifiable markers of the progression of neurodegenerative diseases. Although structural BAGs (derived from structural MRI) have been the most extensively studied to date, emerging evidence suggests that BAGs from different imaging modalities capture distinct aspects of deviation from a typical aging trajectory, including molecular, functional, pathologic, and microstructural brain aging patterns. In the context of neurodegenerative diseases, the (limited) existing data indicate that the choice of imaging modality for BAG estimation may be relevant for assessing disease progression in different neurodegenerative diseases. Structural BAGs, in particular, have shown associations with cognitive decline and AD biomarkers, suggesting their potential as a monitoring tool. Pending further investigation in clinical and real-world settings, BAGs may offer a sensitive, imaging-based tool for identifying individuals at risk, monitoring disease trajectories, and assessing therapeutic impact in neurodegeneration.
